# Supplementary material for: The Impact of Different Antihypertensive Drugs on Cardiovascular Risk in Isolated Systolic Hypertension with Type 2 Diabetes Patients
Source: J Clin Med. 2022 Nov 1;11(21):6486. doi: 10.3390/jcm11216486 (PMC9655533; doi:10.3390/jcm11216486)
Supplement: Supplementary file 1 [file jcm-11-06486-s001.zip › jcm-1968287-supplementary.pdf]

Supplementary Table S1. Risk of total mortality for patients on different antihypertensive therapies compared with those without.

Antihypertensive therapy	Model 1		Model 2		Model 3	
	HR (95% CI)	*P* Value	HR (95% CI)	*P* Value	HR (95% CI)	*P* Value
ARB (+) / ARB (-)	0.70 (0.44, 1.12)	0.142	0.67 (0.42, 1.08)	0.098	0.69 (0.43, 1.11)	0.125
ACEI (+) / ACEI (-)	1.41 (0.84, 2.10)	0.224	1.43 (0.86, 2.14)	0.276	1.47 (0.89, 2.04)	0.137
CCB (+) / CCB (-)	1.04 (0.71, 1.51)	0.859	1.09 (0.74, 1.59)	0.672	1.06 (0.72, 1.56)	0.759
TD (+) / TD (-)	0.95 (0.67, 1.36)	0.791	0.97 (0.68, 1.39)	0.874	1.03 (0.71, 1.49)	0.876

Model 1: Unadjusted. Model 2: Adjusted for baseline age and gender. Model 3: Added race, education, Body Mass Index (BMI), smoking history, systolic and dilated blood pressure, HbA1c level, lipid level (low-density lipoprotein, high-density lipoprotein and triglyceride), therapies, previous heart failure, previous CVD, sulfonylureas, biguanides, meglitinide, alpha-glucosidase inhibitors, thiazolidinediones, regular Insulins and statins to model 2. Abbreviations: ARB, angiotensin receptor blocker; ACEI, angiotensin-converting enzyme inhibitor; CCB, calcium channel blocker; TD, thiazide diuretics; HR, hazard ratio; CI, confidence interval. ARB (+), patients treated with ARB; ARB (-), patients treated without ARB; ACEI (+), patients treated with ACEI; ACEI (-), patients treated without ACEI; CCB (+), patients treated with CCB; CCB (-), patients treated without CCB; TD (+), patients treated with TD; TD (-), patients treated without TD.

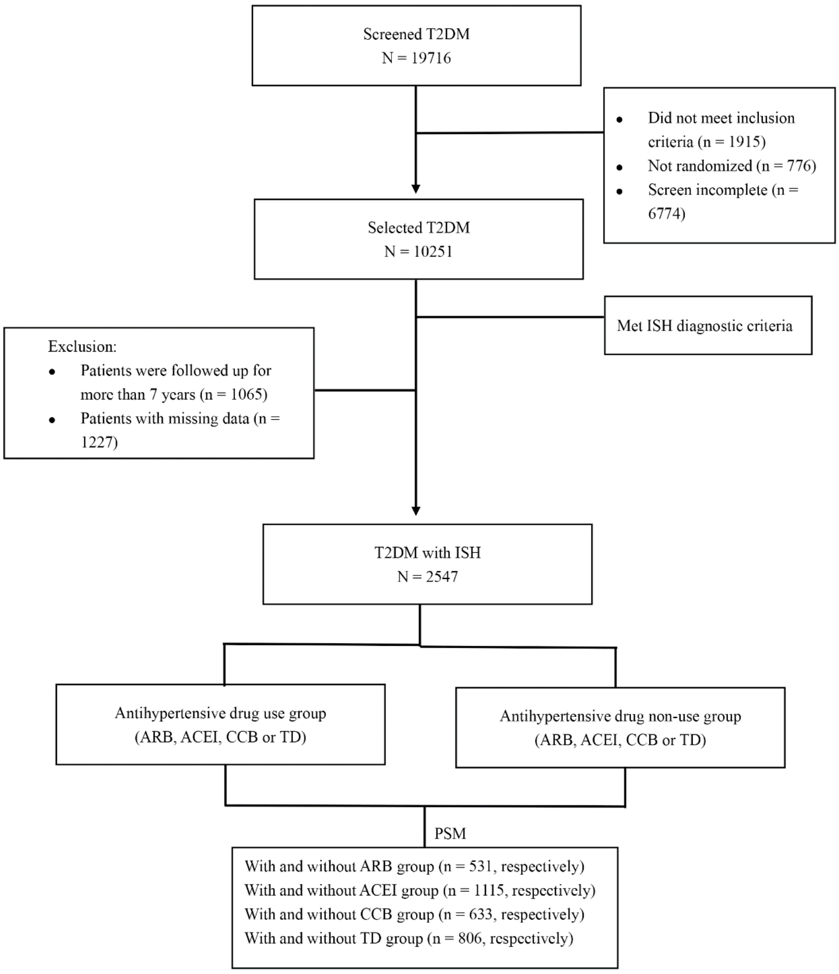

Supplementary Figure S1. A detailed CONSORT-flow diagram of the patients' recruitment.
